# Non-coding RNAs: new biomarkers and therapeutic targets for esophageal cancer

**DOI:** 10.18632/oncotarget.16721

**Published:** 2017-03-30

**Authors:** Xiaobin Hou, Jiaxin Wen, Zhipeng Ren, Guoliang Zhang

**Affiliations:** ^1^ Department of Thoracic Surgery, PLA General Hospital, Beijing, China; ^2^ Medical Science Weekly, Beijing, China

**Keywords:** esophageal cancer, lncRNAs, miRNAs, biomarker, therapeutic target

## Abstract

Esophageal cancer is one of the most common gastrointestinal malignant diseases and there is still no effective treatment. The incidence of esophageal cancer in the world is relatively high and on the increase year by year. Thus, the elaboration on the carcinogenesis of esophageal cancer and the identification of new biomarkers and therapeutic targets is quite beneficial to optimizing the current therapeutic regimen for treating such deadly disease. More and more evidence has shown that non-coding RNAs play an important role in the development and progression of multiple human cancers, including esophageal cancer. microRNAs (miRNAs) and long non-coding RNAs (lncRNAs) are two functional kinds of non-coding RNAs that have been well investigated. They exert tumor suppressive or promoting effect by specifically regulating the expression of certain downstream target genes, which is tumor specific. It is also proved that miRNAs and lncRNAs level in tissue and plasma from esophageal cancer patients are closely correlated with the survival and disease progression, which could be used as a prognostic factor and therapeutic target for esophageal cancer.

## INTRODUCTION

Esophageal cancer is one of the most common gastrointestinal cancers and the incidence is on the increase in recent years [1, 2]. Patients may have unspecific symptoms like wasteness, nausea, weight loss, and the like at early stage, which makes it difficult to be diagnosed. Although great progress has been made on the carcinogenesis and therapy for esophageal cancer, there is still no effective treatment for such deadly disease and the survival remains very poor [3, 4].

Non-coding RNAs are defined as one kind of transcripts that do not encode any protein [5]. So far, several types of non-coding RNAs have been identified by RNA sequencing and bioinformatics analysis. Among them, long non-coding RNAs (lncRNAs) and microRNAs (miRNAs) have attracted great attentions from researchers all over the world. Most miRNAs are deregulated in multiple human diseases including cancers and cancer-related diseases such as cardiovascular disorders [6]. lncRNAs are a new class of non-coding RNAs that are longer than 200 nucleotides [7], and miRNAs are shorter non-coding RNAs with the length of 21-23 nucleotides [8]. It has been proved that lncRNAs could regulate the expression of downstream genes by mediating chromatin, transcriptional and post-transcriptional modification [9], and miRNAs could post-transcriptionally repress the translation of target genes by binding to the untranslated region (UTR) [10]. Plenty of evidence has verified that multiple lncRNAs and miRNAs participate in the development and progression of esophageal cancer, which could exert regulatory effect on a series of biological processes including cell proliferation, cell migration and invasion, and the like [11, 12]. lncRNAs and mRNAs can have crosstalk and work together in regulatory loops [13]. Thus, in this paper we overviewed the recent research advances on the molecular functions of lncRNAs and miRNAs in esophageal cancer, and further discussed their potential roles of early diagnosis and prognostic biomarkers and therapeutic targets, which could help deepen the current understanding and enlighten the future investigations.

## NON-CODING RNAS EXPRESSION PROFILING IN ESOPHAGEAL CANCER

**Figure 1:**
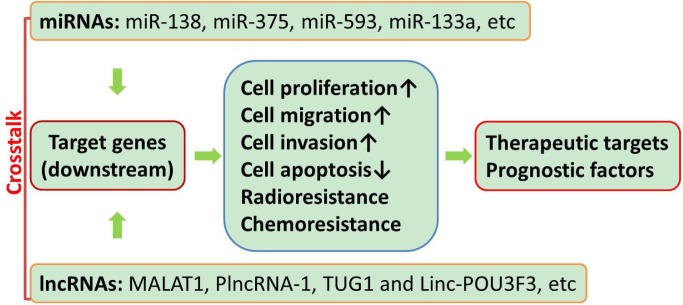
Diagram of the molecular functions of miRNAs and lncRNAs in esophageal cancer Multiple miRNAs and lncRNAs could regulate cell proliferation, migration, invasion, apoptosis, radioresistance and chemoresistance *via* modulating the expressions of target genes in esophageal cancer.

The role of miRNAs and lncRNAs in the pathogenesis of human cancers and other diseases has been elaborated, and there is well-established findings that supports that miRNAs and lncRNAs are very important regulators within human body [14, 15]. They can be either tumor suppressors or tumor promoters [16]. For example, in a cohort of 102 patients who were pathologically diagnosed with esophageal carcinoma, hsa-miR-451a, hsa-miR-144-3p and hsa-miR-144-5p expression in tumor tissues were significantly lower than those in adjacent non-tumor tissues (*P* < 0.05) [17], which were detected by stem-loop reverse transcription-quantitative polymerase chain reaction and bioinformatics tools. Pearson correlation analysis demonstrated that the expression levels of individual miR-144/451 cluster members were correlated with each other, except for hsa-miR-144-3p and hsa-miR-4732-3p. Further analysis demonstrated that hsa-miR-144-5p expression was highly correlated with hsa-miR-4732-5p and hsa-miR-451a expression, and low hsa-miR-144-3p and hsa-miR-144-5p expression was shown to be the independent risk factors for the onset of esophageal carcinoma. Other miRNAs like miR-138, miR-375, miR-593, miR-133a were down-regulated in esophageal cancer tissue, serving as tumor suppressors, while miR-34b, miR-16, miR-208, miR-423, miR-21, miR-31, miR-223 and miR-373 could have oncogenic actions [18–23] (Figure 1). In the same way, lncRNAs also have dual functions. Metastasis-associated lung adenocarcinoma transcript 1 (MALAT1) were down-regulated, while PlncRNA-1, TUG1 and Linc-POU3F3 were up-regulated in esophageal cancer [15, 24–26]. In order to better clarify the expression profiling of non-coding RNAs in esophageal cancer, the database on such patients and tissue collection should be expanded.

## NON-CODING RNAS MOLECULAR FUNCTIONS IN ESOPHAGEAL CANCER

The molecular contributions of non-coding RNAs in the carcinogenesis of esophageal cancer have been widely determined (Table 1). lncRNAs and miRNAs could specifically regulate the expression of certain downstream target genes. Consequently, the dysregulation of these genes are able to play an important role in various physical activity and biological processes and multiple signaling transduction pathways are reported to be involved. Besides, miRNA polymorphisms could disturb the miRNA targetome which was defined as the profile of all the target genes [27], evidenced by in silico experimental data. These miRNAs variants may function in a different manner, which lead to the development of esophageal cancer [28]. However, functional experiments need to be conducted to further confirm it. Based on the recent findings, the molecular functions of lncRNAs and miRNAs in esophageal cancer were summarized below.

**Table 1:** Important non-coding RNAs in human esophageal cancer

Non-coding RNAs	Functions
lncRNA MALAT1	Enhance cell proliferation, G2/M cell cycle arrest, cell migration and invasion
lncRNA PEG10	Increase cell proliferation and migration
lncRNA TP73-AS1	Decrease cell apoptosis and induce chemoresistance
lncRNA CASC9	Increase cell migration and invasion
lncRNA H19	Promote cell proliferation and invasion and induce epithelial-to-mesenchymal transition
miR-373	Enhance cell proliferation, G1-phase cell proportion, migration and invasion
miR-26b	Enhance cell proliferation, cell-cycle transition and migration
miR-100	Inhibit cell proliferation, migration and invasion and suppress tumor growth
miR-98	Restore radiosensitivity
miR-124	Increase cell apoptosis after radiotherapy

### Cell proliferation regulation

lncRNAs (AFAP1-AS1, UCA1, HOTAIR) were found to be dysregulated in cisplatin-resistant esophageal cancer cells compared with the corresponding parent cells [29]. AFAP1-AS1 was significantly up-regulated in tumor tissues compared with adjacent normal tissues. High AFAP1-AS1 level was closely associated with lymph node metastasis, distant metastasis, advanced tumor stage and chemoresponse. lncRNA SPRY4-IT1 level was up-regulated in human advanced esophageal squamous cell carcinoma tissue, which could enhance the cell viability by activating ZNF703 [30]. MALAT1 is a highly conserved long non-coding RNA, and its oncogenic role has been reported in multiple human cancers. MALAT1 could enhance cell proliferation, G2/M cell cycle arrest, cell migration and invasion of esophageal cancer cells by up-regulating p21 and p27 expression and down-regulating B-MYB expression. miR-101, miR-217 and MALAT1 siRNA have common downstream genes like MIA2, HNF4G, ROBO1, CCT4 and CTHRC1, and miR-101 or miR-217 and MALAT1 were negatively correlated in 42 pairs of esophageal cancer tissue samples and adjacent normal tissues [31]. lncRNA PEG10 was overexpressed in esophageal cancer tissues, which was significantly correlated with lymph node metastases. lncRNA PEG10 silencing could decrease cell proliferation and migration, induce cell apoptosis in two esophageal cancer EC9706 and KYSE150 cells [32].

miR-100 was significantly down-regulated in esophageal cancer, and miR-100 overexpression in esophageal cancer cells significantly inhibited cell proliferation, migration and invasion and suppressed tumor growth *via* targeting CXCR7 [33]. miRNAs can also regulate sphere formation cell proliferation in esophageal cancer. STAT3 and miR-181b mutually play an important role in controlling cell proliferation and cell apoptosis resistance of sphere formation cells in esophageal cancer in a positive feedback loop [34]. STAT3 can directly increase miR-181b transcription, which targets CYLD gene and miR-181b can enhance the p-STAT3 activity.

### Cell cycle regulation

Kyoto Encyclopedia of Genes and Genomes pathway analysis indicated that miR-144/451 cluster could regulate the cell cycle, which validates it to be a promising biomarker for early detection of esophageal carcinoma [17]. miR-26b expression was abnormally up-regulated in esophageal cancer cells and human tissue, whereas no significant change was found on miR-26a expression. miR-26b down-regulation could suppress cell proliferation, cell-cycle transition and migration by targeting TRAF5 [35]. MALAT1 silencing could inhibit proliferation-enhanced apoptosis, cell migration/invasion, and reduce colony formation and induce G2/M arrest [24]. MALAT1 overexpression was associated with a decreased survival rate and MALAT1 can be used as a potential therapeutic target for human esophageal squamous cell carcinoma.

### Cell migration and invasion regulation

lncRNA CASC9 was significantly up-regulated in esophageal cancer tissues. CASC9 knockdown could significantly suppress cell migration and invasion, and increased CASC9 expression was associated with cell differentiation *in vitro*, indicating that CASC9 may be identified as a new promising biomarker for poor prognosis and a potential therapeutic target for treating esophageal cancer [36]. lncRNA LOC100130476 up-regulation could suppress cell proliferation and invasion, and hypermethylation of CpG sites in exon 1 could down-regulate LOC100130476 expression in esophageal cancer, which could predict the clinical TNM stage and pathological differentiation [37]. lncRNA H19 was significantly increased and correlated with tumor invasion depth and metastasis. H19 overexpression could promote cell proliferation and invasion and induce epithelial-to-mesenchymal transition, whereas H19 knockdown could inhibit cell proliferation and invasion and reverse epithelial-to-mesenchymal transition *in vitro* [38]. miR-373 has different functions among different tumor types. miR-373 expression was up-regulated in esophageal cancer tissues and patients’ plasma. miR-373 up-regulation could enhance cell proliferation, G1-phase cell proportion, migration and invasion, while miR-373 silencing could decrease cell proliferation, G1-phase cell proportion, migration and invasion and induce cell apoptosis by directly targeting TIMP3. Overexpression of miR-373 in ECA109 caused a reduction of TIMP3 mRNA and protein, whereas suppression of miR-373 in KYSE410 led to an increase of TIMP3 mRNA and protein [39].

### Cell apoptosis regulation

lncRNA TP73-AS1 and its target gene BDH2 were both up-regulated and closely correlated with the tumor location and TNM stage in esophageal cancer tissues. lncRNA TP73-AS1 knockdown inhibited BDH2 expression in EC9706 and KYSE30 cells, which could further inhibit esophageal cancer cell proliferation and induce cell apoptosis *via* the caspase-3 dependent apoptotic pathway both *in vitro* and *in vivo*. BDH2 modulation could partially rescue the effect of lncRNA TP73-AS1 on cell proliferation and apoptosis [40]. lncRNA AFAP1-AS1 was hypomethylated and overexpressed in Barrett's esophagus and esophageal cancer. AFAP1-AS1 silencing by siRNA could inhibit cell proliferation, migration and invasion, suppress colony-forming ability and induce cell apoptosis, but the protein-coding counterpart AFAP1 was not affected [41]. Based on miRNAs profiling data, miR-20b, miR-498 and miR-196 were predicted to be involved in cell apoptosis and autophagy, which are shown to be key regulators of multiple cellular signaling pathways in esophageal cancer [42].

### Chemosensitivity regulation

Chemoresistance is the key to the achievement of effective esophageal cancer therapy. miRNA-127-3p, which specifically compromised the homologous recombination repair and significantly increased DNA double strand breaks in cells, could statistically increase the chemosensitivity of esophageal cancer cells to a novel phenanthroline-dione derivative *in vivo* by mechanistically impairing the recruitment of RAD51 to the damage sites [43]. miR-221 was overexpressed in 5-fluorouracil resistant esophageal cancer cells and human esophageal cancer tissue. miR-221silencing could reduce cell proliferation, increase apoptosis and chemosensitivity, and inactivate the Wnt/β-catenin pathway mediated by alteration in DKK2 expression both *in vitro* and *in vivo* [44]. BDH2 or lncRNA TP73-AS1 knockdown enhanced the chemosensitivity of esophageal cancer cells to and cisplatin. Our results suggest that lncRNA TP73-AS1 may be a novel prognostic biomarker that could serve as a potential therapeutic target for the treatment of esophageal cancer [40]. circRNAs dys-regulation, which mainly refer to serum and plasma RNAs were found in human radioresistant esophageal cancer cells, and CircRNA_001059 and circRNA_000167 were the two largest nodes to form a comprehensive and functional circRNA/miRNA co-expression network [45]. LOC285194 expression was significantly down-regulated in esophageal cancer. Low LOC285194 expression was correlated with tumor size, TNM stage, lymph node metastases and distant metastases. Univariate and multivariate analysis demonstrated that LOC285194 down-regulation could predict poor chemotherapy response and survival status, evidenced by the fact that patients with low LOC285194 level had a decreased disease free survival and overall survival [46].

### Radiosensitivity modulation

Aberrant miRNAs expression is also responsible for impairing radiosensitivity. miR-98 was down-regulated in radioresistant esophageal cancer cell line, and miR-98 mimic could restore the sensitivity to radiotherapy in esophageal cancer cells by up-regulating miR-98, which could decrease cell proliferation and migration and induce cell apoptosis by directly binding to the promoter of BCL-2 gene [47]. miR-124 expression was reduced in esophageal cancer tissue, and miR-124 overexpression could increase the percentage of apoptotic cells following radiotherapy by targeting CDK4 in esophageal cancer TE-1 cells [48].

## POTENTIAL BIOMARKERS AND THERAPEUTIC TARGETS FOR HUMAN ESOPHAGEAL CANCER

### Diagnostic and prognostic biomarkers

Detection of miRNAs and lncRNAs could be introduced as the potential biomarkers for early diagnosis and prognosis [49–51]. A microarray analysis has ever identified miR-574-3p, miR-106b, miR-1303, miR-1203, miR-1909, miR-204, miR-371-3p and miR-886-3p, which were differentially expressed between the patients with and without tumor relapse after surgery. Higher expression of miR-574-3p was associated with non-relapse and favorable overall survival, which was a predictor for clinical outcome in patients with esophageal cancer after surgery [18]. Univariate and multivariate analysis revealed that high pretreatment plasma miR-21 level was an independent risk factor for chemoresistance in esophageal cancer, which was greatly correlated with high histopathological response [52]. miR-21 level was significantly elevated in esophageal cancer, while miR-375 was down-regulated. miR-21 overexpression could reduce the radiosensitivity and increase the tumor relapse. The combination of miR-21 and miR-375 level was identified to be a biomarker for early diagnosis and prognosis of esophageal cancer [19].

Survival analysis revealed that high lncRNA AFAP1-AS1 level was significantly associated with shorter progression free survival and overall survival and multivariate analysis showed that high AFAP1-AS1 level was found to be an independent risk factor for poor clinical response [29]. Hypermethylation of CpG sites in the exon 1 and low expression of LOC100130476 could predict poor survival [37]. HOTAIR overexpression was correlated with short survival in esophageal cancer regardless of the ethnicities, indicating that high HOTAIR level may be a prognostic factor for esophageal cancer [53]. lncRNA CCAT2 was up-regulated in esophageal cancer tissues and positively correlated with TNM stage and lymph node metastasis. Survival analyses revealed that high CCAT2 expression and MYC amplification were significantly associated with poorer overall survival in such patients. Patients with high CCAT2 expression and MYC amplification had an increased risk of cancer-related death, supporting that CCAT2 is a predictive biomarker and therapeutic target for esophageal cancer [54]. lncRNA PCAT-1 was significantly higher in human esophageal cancer, and high PCAT-1 expression was significantly correlated with advanced clinical stage, lymph node metastasis, and poor prognosis, which is a potential prognostic factor for esophageal cancer [55]. Now, the detection of plasma lncRNAs identified POU3F3, HNF1A-AS1 and SPRY4-IT1 were greatly higher in esophageal cancer patients. Among them, plasma POU3F3 had the highest diagnostic performance for esophageal cancer and the combination of POU3F3 and SCCA had a more excellent diagnostic efficiency, especially for early stage [56]. lncRNA UCA1 was overexpressed in esophageal cancer cells and was prone to predict poor prognosis. Targeting UCA1 could decrease cell proliferation, migration and invasion [57]. A study has ever analyzed the associations of lncRNAs with the risk and prognosis of esophageal cancer in 358 patients from eastern China, and validate the findings in another 326 patients from southern China. lncRNA uc002yug.2 was overexpressed in esophageal cancer, which could exert oncogenic effect by overexpressing RUNX1a and reducing CEBPα gene expression. These data suggest that lincRNA-uc002yug.2 level could be a prognostic factor for the esophageal cancer patients’ survival [58].

lncRNAs can also co-function with the adjacent coding genes by forming a lncRNA-mRNA gene pair. For example, lncRNA FOXCUT and its neighboring gene FOXC1. FOXCUT and FOXC1 levels were both up-regulated in esophageal cancer and strongly correlated with poor differentiation and metastasis. Up-regulated FOXCUT or FOXC1 expression could predict a significantly worse prognosis. FOXCUT level was positively correlated with FOXC1 level in esophageal cancer, and the decreased expression of FOXC1 was also observed after being treated by FOXCUT siRNA. *In vitro* experiments showed that either FOXCUT or FOXC1 silencing could greatly inhibit cell proliferation, colony formation, migration and invasion in esophageal cancer cells. FOXCUT plays a functional role in the development of esophageal cancer and predicts the survival potentially and partially by modulating FOXC1 [59]. Besides, a recent study identified a three lncRNA signature (including ENST00000435885.1, XLOC_013014 and ENST00000547963.1), which was proved to be able to independently predict the outcome of the patients [60].

### Therapeutic targets

miR-100 functions as a tumor suppressor in esophageal cancer, which could be potentially applied in treating esophageal cancer [33]. The miR-124/CDK4 axis was an important mechanism in regulating the radiation sensitivity of human esophageal cancer cells, and targeting CDK4 may improve the clinical efficacy of radiotherapy [48]. lncRNA BOKAS was up-regulated and increased WISP1 expression, which could lead to the resistance to radiotherapy in esophageal cancer both *in vitro* and *in vivo*. WISP1 was a oncofetal gene in Wnt/β-catenin pathway, and WISP1 level could predict the prognosis of esophageal cancer patients after radiotherapy. Molecular investigation indicated that WISP1 could contribute to radioresistance *via* repairing irradiation-induced DNA damage and activating PI3K kinase and its function was in a positive feedback loop [61]. Previous researches reported that β-elemene could inhibit cell proliferation of esophageal cancer ECA-109 cells and up-regulate about 85% of the lncRNAs except CDKN2B-AS1. Targeting the β-elemene-mediated lncRNA CDKN2B-AS1 decreased cell proliferation, and increased cell apoptosis by down-regulating the hTERT level [62]. lncRNA HNF1A-AS1 was highly up-regulated in human primary esophageal cancer tissues. HNF1A-AS1 silencing significantly inhibited cell proliferation and anchorage-independent growth, S-phase entry, cell migration and invasion *in vitro* by preferentially modulating genes that are linked to assembly of chromatin and the nucleosome, which is a key mechanism essential to cell cycle progression. lncRNA HNF1A-AS1 silencing could also inhibit lncRNA H19 expression. Consistent to this finding, HNF1A-AS1 could be a very important therapeutic target for treating esophageal cancer [63]. lncRNA PEG10 up-regulation could suppress cell proliferation, cell invasion and migration in esophageal cancer, which is qualified to be a promising therapeutic target in esophageal cancer [32].

## CONCLUSIONS

Multiple miRNAs and lncRNAs play an important role in the development and progression of esophageal cancer, which could be identified to be new promising therapeutic targets and prognostic factors. However, the current findings mainly focus on the basic research on the functions of non-coding RNAs in esophageal cancer. Great progress should be made to fully understand the clinical application of miRNAs and lncRNAs in diagnosing and treating such devastating disease. Targeting specific downstream genes by miRNAs and lncRNAs in esophageal cancer will be a promising therapeutic regimen, which should be further validated in future studies.
